# Rate dependent influence of arterial desaturation on self-selected exercise intensity during cycling

**DOI:** 10.1371/journal.pone.0171119

**Published:** 2017-03-03

**Authors:** Saro D. Farra, Stephen S. Cheung, Scott G. Thomas, Ira Jacobs

**Affiliations:** 1 Faculty of Kinesiology & Physical Education, University of Toronto, Toronto, Ontario, Canada; 2 Department of Kinesiology, Brock University, St. Catherines, Ontario, Canada; Universidad Europea de Madrid, SPAIN

## Abstract

The purpose of this study was to clarify if Ratings of Perceived Exertion (RPE) and self-selected exercise intensity are sensitive not only to alterations in the absolute level of arterial saturation (S_P_O_2_) but also the rate of change in S_P_O_2_. Twelve healthy participants (31.6 ± 3.9 y, 175.5 ± 7.7 cm, 73.3 ± 10.3 kg, 51 ± 7 mL·kg^-1^·min^-1^ $\dot{V}O_{2\mathrm{peak}}$) exercised four times on a cycle ergometer, freely adjusting power output (PO) to maintain RPE at 5 on Borg’s 10-point scale with no external feedback to indicate their exercise intensity. The fraction of inspired oxygen (F_I_O_2_) was reduced during three of those trials such that S_P_O_2_ decreased during exercise from starting values (>98%) to 70%. These trials were differentiated by the time over which the desaturation occurred: 3.9 ± 1.4 min, -8.7 ± 4.2%•min^-1^ (FAST), 11.0 ± 3.7 min, -2.8 ± 1.3%•min^-1^ (MED), and 19.5 ± 5.8 min, -1.5 ± 0.8%•min^-1^ (SLOW) (P < 0.001). Compared to stable PO throughout the control condition (no S_P_O_2_ manipulation), PO significantly decreased across the experimental conditions (FAST = 2.8 ± 2.1 W•% S_P_O_2_^-1^; MED = 2.5 ± 1.8 W•% S_P_O_2_^-1^; SLOW = 1.8 ± 1.6 W•% S_P_O_2_^-1^; P < 0.001). The rates of decline in PO during FAST and MED were similar, with both greater than SLOW. Our results confirm that decreases in absolute S_P_O_2_ impair exercise performance and that a faster rate of oxygen desaturation magnifies that impairment.

## Introduction

There are a myriad of mediators that have been associated with the development of fatigue during voluntary exercise such as increasing core body temperature (T_C_) [1, 2], accumulating concentrations of muscle and blood metabolites [3, 4], energy availability [5–7], and insufficient oxygen (O_2_) availability and/or transport [8, 9]. The research conducted to date in the area of exercise-induced fatigue demonstrates how the magnitude of changes in various ambient stressors, or the absolute magnitude of physiological strain associated with those stressors, augment the development of exercise-induced fatigue. Much less is known about how the rate of change of these same stressors and strains influence the homeostatic control systems that are thought to regulate the neuromuscular system during exercise. Should the rate of change in physiological strain be shown to influence exercise performance, it would support the proposition that the neuromuscular system is sensitive to feed-forward homeostatic control during exercise [10, 11], because feed-forward systems can be characterized as being able to differentiate between the instantaneous physiological state and the instantaneous rate of change within the system [12, 13].

It has been suggested that the feed-forward regulation of voluntary exercise performance is based on one’s ratings of perceived exertion (RPE) [14], which is defined as the conscious manifestation of the degree of strain experienced during physical work [15]. Although several theories exist regarding the physiological and psychological determinants of RPE [16], research suggests that it is an integration of multiple sources of information including corollary discharge of the efferent output from the motor cortex [17–20], afferent feedback from the periphery [21–26], as well as psychological factors such as previous experience and knowledge of exercise duration [27], motivation [16], positive and negative affect [28] and awareness [29]. Although isolating the feed-forward contribution to voluntary exercise performance can be challenging, Tucker [11] proposed a conceptual model, known as ‘constant RPE exercise’, that may be useful to further this line of research. In this model, the investigator dictates a target RPE (known as the RPE template), and the subject then maintains that RPE by freely adjusting exercise intensity. According to this model, the control of exercise intensity is the result of error correction feedback, as oscillations in exercise performance occur as the central nervous system (CNS) attempts to minimize the difference between the conscious RPE and the ‘RPE template’. When participants exercise at a moderate intensity, they reliably reproduced the exercise intensity and the associated physiological steady state throughout treadmill [30] and cycling [26, 31] exercise in the absence of any knowledge about the exercise intensity or their physiological responses [26, 30, 31]. Other forms of self-paced exercise, such as a time trial does not allow for this consistency, as exercise intensity and the associated physiological responses are constantly changing. Such a consistent and reproducible steady state over the duration of an exercise trial lends itself to experimental manipulation seeking to separate the impact of the absolute magnitude of physiological strain from the rate of physiological strain development on exercise performance as the manipulation can be applied irrespective of time.

It is well established that exercise performance is impaired in environments where oxygen (O_2_) supply is reduced. Exposure to hypoxia is associated with centrally mediated reductions in muscle activation [32, 33], and the mechanisms responsible for this central impairment shift with increasing hypoxia severity. While exercising in mild hypoxia (F_I_O_2_ = 0.15 to 0.16) [32, 34] increasing levels of afferent feedback from fatiguing muscles have been proposed to limit central motor drive (CMD) [3, 35] so that the development of peripheral muscle fatigue does not surpass a critical threshold [35, 36]. In this theoretical framework, hypoxia exacerbates the accumulation of metabolites that stimulate group III & IV afferents [37], triggering inhibitory sensory feedback to the central nervous system (CNS). While exercising in severe hypoxia (F_I_O_2_ = 0.10), the hypoxic stress diminishes the pressure gradient for gas exchange between the lungs and the active tissues [38], which may interfere with neurotransmitter turnover [39] or impair cerebral aerobic metabolism [40–42]. However, the supporting research, for both mild and severe hypoxia, reports how absolute changes in hypoxic stress impaired exercise performance and not whether the rate of change in either hypoxic stress and/or strain may modulate self-selected exercise intensity. Some research speculates that self-selected exercise intensity is sensitive to the rate of physiological strain development [43–45]. With respect to traditional models of exercise-induced fatigue accompanying arterial hypoxemia, it is unclear how and why the rate of physiological strain development would influence exercise performance [38, 46–48]. However, newer theories suggest that the CNS uses both the absolute magnitude and the rate of change in physiological strain as independent modulators of self-selected exercise intensity [11]. The mechanism of this proposed form of regulation remains currently unknown.

Therefore, the aim of this study was to investigate how different rates of arterial desaturation impact self-selected exercise intensity. As previous research suggests that the rate of physiological strain development influences exercise performance, this study was designed to test the hypothesis that faster rates of arterial deoxygenation will augment the declines in muscle activity and self-selected power output (PO) while cycling at a fixed RPE, as well as the peak power output (PPO) generated during a 5 s maximal effort sprint.

## Materials and methods

### Participants

Twelve participants (8 male and 4 female) volunteered to participate in this study. Sample size calculations were planned to detect a 15 W difference in our main outcome variable, PO at an RPE of 5 on Borg’s 10-point scale. Based on a within-subject standard deviation of 11 W (determined during pilot testing), this sample size was required to detect a treatment difference at the 0.05 level, with a power of 0.80. Participants were healthy, competitive cyclists. The mean physical and physiological characteristics are listed in Table 1. Prior to their commencement, participants were informed of the risks associated with the study and a written informed consent was obtained. Participants were asked to avoid ingesting any food or drink (besides water) for 2 h before testing, to refrain from consuming caffeinated food or beverages for 12 h before testing, and not to exercise for 24 h before any testing session. The Health Sciences Research Ethics Board of the University of Toronto approved the protocol for this study. All experiments conformed to the standards set by the Declaration of Helsinki.

**Table 1:** Physical and physiological subject characteristics.

	Age	Height	Weight	P_max_	$\dot{V}O_{2\mathrm{Peak}}$	HR_max_	${\dot{V}}_{\mathrm{Emax}}$
	(y)	(cm)	(kg)	(W)	(ml•kg^-1^•min^-1^)	(BPM)	(L•min^-1^)
Male	31.3 ± 3.7	179.9 ± 3.4	79.1 ± 6.2	330 ± 32	53 ± 5	190 ± 6	161 ± 24
Female	30.2 ± 5.2	166.7 ± 5.7	61.8 ± 6.1	215 ± 17	46 ± 4	181 ± 11	133 ± 27
Group	30.9 ± 4.1	175.5 ± 7.7	73.3 ± 10.4	293 ± 63	51 ± 6	187 ± 8	154 ± 27

P_max_, Highest exercise intensity achieved during the incremental step test to exhaustion

### Protocol

Participants reported to the laboratory on six occasions, with sessions being separated by at least 48 h. Throughout the study, all exercise was preceded by a 15 min warm up on the cycle ergometer at a self-chosen intensity corresponding to a RPE of 5 on Borg’s 10-point scale. During the first visit, basic physiological and physical characteristics were measured, including height, weight, and peak aerobic power ($\dot{V}O_{2\mathrm{peak}}$) during cycle ergometry. Participants began the incremental test at 90 W. The workload increased in a step-wise fashion by 30 W every 3 min until 1) the investigators terminated the test because participants were not able to maintain their cadence within 20 RPM of their self-selected target for more then 30 s, or 2) the participant reached volitional fatigue. During the test, participants were connected to a metabolic cart that measured respiratory parameters on a breath-by-breath basis. $\dot{V}O_{2\mathrm{peak}}$ (ml^.^kg^-1.^min^-1^) was defined as the highest 30 s average O_2_ consumption that was recorded during the test.

During the second visit, the relationship between the partial pressure of inspired O_2_ (P_I_O_2_) and arterial saturation (S_P_O_2_) was modeled under isocapnic conditions.

During the four remaining visits, the study utilized a single-blind, crossover design where experimental and control trials were completed in random order. Participants cycled on an isokinetic cycle ergometer at an intensity they felt corresponded to a RPE of 5 on Borg’s 10-point scale [25] for 30 min, with the final stage of the $\dot{V}O_{2\mathrm{peak}}$ test as their anchor for what would constitute a “10”; effort was adjusted with fatigue, such that subjective sensation of whole body exertion remained steady at target level of 5 [45]. On three trials, the fraction of inspired O_2_ (F_I_O_2_) was reduced to desaturate arterial blood from starting values (>98%) to 70% while maintaining isocapnia. This desaturation was designed to occur approximately linearly over 3 different time periods: 5 min, 15, min, and 25 min for the FAST, MED, and SLOW conditions respectively. When S_P_O_2_ reached 70%, F_I_O_2_ was surreptitiously switched back to 21% for the remainder of the 30 min trial. During the control trial (CON), F_I_O_2_ remained at 21%. Participants were blinded to P_I_O_2_ and were kept naive as to the hypotheses of the study. More specifically, participants were made aware of the absolute level of arterial desaturation they were to undergo, but were not informed that different desaturation rates would be used during each of the trials.

Participants also performed several 5 s maximal effort sprints to measure PPO at the following intervals: two sprints prior to commencing the constant RPE trial (PRE), one sprint at a S_P_O_2_ of 70% (POST), and two sprints 2 min after POST when S_P_O_2_ recovered back to initial values (REC). During PRE and REC, each sprint was separated by 3 min and the trial with the highest PPO was selected for analysis. For some participants, the constant RPE trials were terminated prior to achieving a S_P_O_2_ of 70%, when the subject indicated that they wished to stop the trial, or when the investigator observed the participant displaying symptoms that indicated severe hypoxic strain. The participants that were able to achieve a S_P_O_2_ of 70% did so repeatedly across conditions. However, when a participant did not achieve a S_P_O_2_ of 70% during the first hypoxic trial, the investigator terminated all subsequent constant RPE exercise trials so that the final S_P_O_2_, and the S_P_O_2_ during the 5 s sprints, would be similar across conditions. All participants were made aware of the termination criteria.

### Hypoxia administration

#### Isocapnic hypoxia system

The isocapnic hypoxia system (IHS), developed in our laboratory [49], was used to decrease S_P_O_2_ while clamping the partial pressure of end-tidal carbon dioxide (PET_CO2_) within 2 mmHg of starting values throughout and in between all exercise trials. Isocapnia was maintained utilizing the principles of sequential gas delivery [50–52]. Briefly, the IHS included a 3-way breathing manifold connected to an inspiratory and expiratory reservoir. The configuration of the manifold was such that it first delivered fresh gas from the inspiratory reservoir, and when this source of gas was depleted, it sequentially delivered previously expired gas from the expiratory reservoir. The flow of fresh gas into the inspiratory reservoir, as well as its composition, were controlled by custom computer software (LabView, National Instruments, Austin, TX) regulating two independent mass flow controllers (Model # 32907–77, Cole-Parmer, Montreal), with each mass flow controller adjusting the flow of one source gas (Tank 1–21% O_2_, balance nitrogen [N_2_]; Tank 2–5% O_2_, balance N_2_). Throughout the trial, the investigator adjusted the gas flow rate manually and the software automatically regulated changes in gas composition according to user-defined parameters as indicated below. At the start of each trial, the investigators monitored PET_CO2_ and adjusted the rate of fresh gas delivery as required to clamp PET_CO2_ during the exercise trials.

#### Modeling the P_I_O_2_-S_P_O_2_ relationship

Participants were connected to the IHS and cycled at an intensity that was associated with their first ventilatory breakpoint, determined during the $\dot{V}O_{2\mathrm{peak}}$ test [53, 54]. Participants exercised at this intensity under isocapnic conditions, while F_I_O_2_ levels were decreased every 3 min (21%, 18%, and 14%). S_P_O_2_ was recorded at the end of each stage. F_I_O_2_ was multiplied by barometric pressure to calculate P_I_O_2_ (mmHg). The relationship between P_I_O_2_ and S_P_O_2_ was individually modelled for each subject with a second order polynomial function. Eq 1 exemplifies this function for one subject:

$$P_{I}O_{2}=0.12x^{2}-16.5x+679.5$$ (1)

where *x* = *S*_*P*_*O*_2_ (%).

#### Progressive arterial desaturation

During the first 5 min of the four constant RPE trials, the IHS was supplied with 21% O_2_ so that participants could establish a level of voluntary effort that corresponded to the target RPE. At the start of the of the desaturation procedure, the F_I_O_2_ of the inspirate was continuously reduced over time according to a second order polynomial function (Eq 2). For each experimental condition, a different function was developed to decrease S_P_O_2_ linearly over the target duration of the trial (Eq 2), according to the P_I_O_2_-S_P_O_2_ relationship (Eq 1) previously established. Eqs 2A–2C exemplify these functions for one subject:

$$P_{I}O_{2}=4.1x^{2}-36.2x+157.2$$ (2A, FAST)

$$P_{I}O_{2}=0.5x^{2}-12.1x+157.2$$ (2B, MED)

$$P_{I}O_{2}=0.2x^{2}-7.2x+157.2$$ (2C, SLOW)

where *x* = *Time* (*min*).

#### Data acquisition

All data were synchronously recorded (Power lab 16/35, ADInstruments, Australia), digitized and stored on a laptop computer for later analysis.

#### S_P_O_2_ and Heart Rate (HR)

S_p_O_2_ and HR were sampled at 1 Hz using a pulse oximeter (Model # 7500, Nonin Medical, USA). The left earlobe was prepared by applying hyperemic cream (Finalgon, Boehringer-Ingelheim, France) for 8 min. The probe was attached to the earlobe after cleaning the area with isopropyl alcohol.

#### Cycle ergometry

All cycling exercise was performed on an electronically braked cycle ergometer (Excalibur Sport, Lode, Groningen, Netherlands), which sampled PO at 5 Hz. This ergometer can be used for exercising at a specific absolute PO within a broad range of pedaling frequencies (hyperbolic mode), or it can be used to regulate the rate of pedaling (RPM) at a target cadence and register the power that is being generated (isokinetic mode). During the $\dot{V}O_{2\mathrm{peak}}$ test the ergometer was set to its hyperbolic mode. During all constant RPE exercise trials and 5 s sprints, the ergometer operated in its isokinetic mode where participants self-selected their preferred RPM. The power that they chose to generate was recorded continuously and participants were required to complete all subsequent cycling exercise at that cadence. Because the ergometer regulated cadence at a set rate, participants could only alter PO by increasing or decreasing their force on the pedals to maintain RPE at a 5 of Borg’s 10-point. No visual or verbal feedback was provided except for the occasional reminder to sustain a consistent perceived effort throughout the trial. The participants used the same footwear and pedals for every session. Each subject’s set-up was recorded in the first session and was subsequently reproduced for the remaining tests.

#### Surface electromyography

Surface electromyography (sEMG) signals from the vastus medialis (VM) and vastus lateralis (VL) were measured via bipolar Ag-Ag-Cl surface electrodes (inter-electrode distance 2 cm). Signals were amplified with an isolated differential amplifier (Dual Bioamp FE 135, ADInstruments, Australia), band pass filtered (10–500Hz), and digitized with a sampling frequency of 2000 Hz. Electrodes were positioned over the muscle belly along the assumed angle of pennation after shaving and cleaning the area with isopropyl alcohol. Electrodes and wires were first anchored using adhesive tape (Ref # 71443–02, Hypafix, Germany), which was than covered with a mesh sleeve (Cat # GL-705, Surgilast, Canada) to reduce movement artifact. sEMG signals for both muscles were quantified by Root-Mean-Square (sEMG_RMS_). During constant RPE exercise, sEMG_RMS_ values were normalized to the initial 30 s average value when S_P_O_2_ was roughly 100%. During the 5 s sprint sEMG_RMS_ were normalized to PRE values.

#### Near infrared spectroscopy

Indicators of cerebral and muscle oxygenation were measured with near infra-red spectroscopy (NIRS) (Niro 300, Hamamatsu, Japan) with a sampling rate of 1 Hz. NIRS measured tissue oxygenation index (TOI) and oxy-hemoglobin (O_2_Hb) in response to arterial desaturation. The cerebral probe was positioned over the left pre-frontal cortex (PFC) 1 cm above the eyebrow and 1 cm to the left of the skull centre [55]. The muscle probe was positioned over the right VL along the vertical axis of the thigh, approximately 10–14 cm from the knee joint [56]. The probes were fixed using a dense rubber vinyl holder and held in place with adhesive tape (Ref # 71443–02, Hypafix, Germany) and covered with a mesh sleeve (Cat # GL-705, Surgilast, Canada). Measures are expressed as relative changes from the start of the trial.

#### Minute ventilation

${\dot{V}}_{E}$ was determined on a breath-by-breath basis using a heated pneumotach (Model # 3813, Hans Rudolph, USA) positioned on the expiratory limb of the sequential gas delivery breathing manifold. Airflow was sampled at 100 Hz and signals were low pass filtered (1 Hz). ${\dot{V}}_{E}$ was calculated as:

$${\dot{V}}_{E}=\frac{V_{T}}{\left(T_{B}/60\right)}$$ (3)

where,

$$\begin{array}{l} V_{T}=Tidal\;Volume\;(L)=integral\;of\;flow-time\;signal\;and,\\ T_{B}=Breathing\;Time\;(s)=time\;between\;the\;start\;of\;two\;successive\;expirations. \end{array}$$

*Partial pressure of O*_*2*_ *and CO*_*2*_: The partial pressure of O_2_ and CO_2_ were continuously measured at 100 Hz using a gas analyzer (O2CapB, Oxigraf, USA) sampling near the mouth. Inspired and end-tidal values for O_2_ and CO_2_ were determined on a breath-by-breath basis using the built in “cyclic measurements” functions of our data acquisition software (LabChart V8.0 Pro, ADInstruments, Australia).

### Data processing

To analyze decrements in self-selected PO relative to reductions in S_P_O_2_, 15 s averages were calculated for PO and S_P_O_2_. To estimate the time delay between the start of arterial desaturation and the subsequent decrease in PO [57], the point at which PO in each hypoxic trial began to deviate relative to CON was determined by visual inspection. To determine the slopes of the S_P_O_2_ vs. time, PO vs. S_P_O_2_ and sEMG_RMS_ vs. S_P_O_2_ relationships using linear regression, 5 s averages were calculated for S_P_O_2_, PO, and sEMG_RMS_. To illustrate the change in HR, ${\dot{V}}_{E}$, and NIRS responses over the course of the constant RPE exercise bout, 30 s averages were calculated at defined stages during the trial. INITIAL represents the average of the first 30 s, while FINAL denotes the average of the last 30 s. For the three experimental conditions, MIDDLE represent the 30 s average at a S_P_O_2_ of 85% (midpoint with respect to S_P_O_2_), while for CON it denotes the midpoint with respect to time. During the 5 s sprints, PPO was calculated as the average PO over the 5 s effort, while the difference in sEMG_RMS_ between POST and PRE as well as REC and PRE were calculated.

### Statistical analysis

Results are presented as mean values ± standard deviation (SD). Differences were considered significant when P < 0.05. A one-way repeated measures analysis of variance (RM-ANOVA) was performed to evaluate the differences in the slope of the S_P_O_2_ vs time, PO vs S_P_O_2_, and sEMG_RMS_ vs. S_P_O_2_ between the experimental conditions. All other responses during the constant RPE trials were analyzed using a two-way RM-ANOVA (Condition: *FAST*, *MED*, *SLOW*, *CON* X Stage: *INITIAL*, *MIDDLE*, *FINAL*). Four participants voluntarily stopped some exercise trials prior to achieving a S_P_O_2_ of 70%. Therefore PO at 70% S_P_O_2_ was not included in that analysis. Responses during the 5 s effort were analyzed using a two-way RM-ANOVA (Condition: *FAST*, *MED*, *SLOW*, *CON* X S_P_O_2_: *POST*, *REC*). When the assumption of sphericity was violated, a Greenhouse-Geisser correction was used. Significant findings were followed up with *post hoc* pairwise analysis using a Bonferroni adjustment. All statistics were calculated using SPSS software (Version 22 for Mac, IBM).

## Results

### Arterial saturation

**Fig 1:**
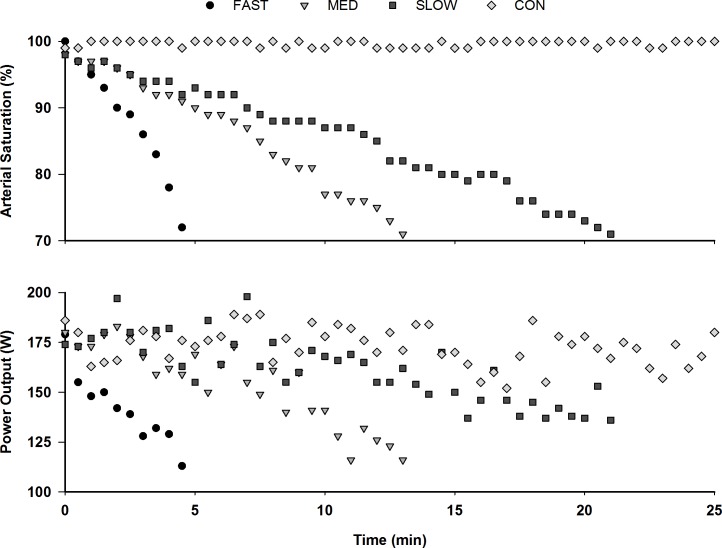
Representative data from one subject illustrating changes in S_P_O_2_ (%) over time (min) (top) and PO (W) over time (min) (bottom). Constant RPE exercise was performed for 30 min. The subject was breathing normoxic gas for the first 5 min during the exercise period so that they could establish a level of voluntary effort that corresponded to a RPE of 5. The desaturation procedure was initiated at 0 min, which was the official start of the experimental trials.

S_P_O_2_ was at or above 98% at the start of each exercise trial (FAST = 98 ± 1%; MED = 98 ± 1%; SLOW = 98 ± 1%; CON = 100 ± 1%). S_P_O_2_ remained above 98% throughout CON, which lasted 22.7 ± 3.7 min. Technical equipment difficulties caused early termination of exercise during CON for some subjects before completing the full trial. S_P_O_2_ decreased from starting values to approximately 70% (or the lowest tolerable S_P_O_2_; three participants reached 75%; one participant achieved 80%), in 3.9 ± 1.4 min, 11.0 ± 3.7 min, 19.5 ± 5.8 min for the FAST, MED, and SLOW conditions respectively, with the rate of arterial desaturation significantly different between the conditions (Table 2)(P < 0.001). Representative data from one subject, illustrating the S_P_O_2_ vs. time response, are shown in Fig 1 (top).

**Table 2:** Rates of change during constant RPE exercise trials for FAST, MED, and SLOW. S_P_O_2_ remained at or above 98% throughout CON.

	FAST	MED	SLOW
S_P_O_2_ (%•min^-1^)	-8.7 ± 4.2^1^^,^ ^2^^,^ ^3^	-2.8 ± 1.3^1^^,^ ^2^	-1.5 ± 0.8^1^
PO (W•%S_P_O_2_^-1^)	2.8 ± 2.1^2^	2.5 ± 1.8^2^	1.8 ± 1.6
VM sEMG_RMS_ (%•%S_P_O_2_^-1^)	1.3 ± 0.6^2^	1.1 ± 0.5^2^	0.7 ±0.7
VL sEMG_RMS_ (%•%S_P_O_2_^-1^)	1.2 ± 0.6^2^	0.9 ± 0.6	0.6 ± 0.5
VL TOI (%•%S_P_O_2_^-1^)	0.3 ± 0.1	0.3 ± 0.1	0.3 ± 0.1
VL O_2_Hb (%•%S_P_O_2_^-1^)	0.2 ± 0.1	0.2 ± 0.1	0.2 ± 0.1
PFC TOI (%•%S_P_O_2_^-1^)	0.4 ± 0.2	0.4 ± 0.2	0.4 ± 0.1
PFC O_2_Hb (%•%S_P_O_2_^-1^)	0.3 ± 0.1	0.4 ± 0.1	0.3 ± 0.1

Values are means ± SD; n = 12

^1^ Significantly different from CON (P < 0.05)

^2^ Significantly different from SLOW (P < 0.05)

^3^ Significantly different from MED (P < 0.05)

### Power output

The average cadence selected by the participants was 91 ± 6 RPM. There was a significant interaction between S_P_O_2_ and condition on PO during the constant RPE trials (P = 0.002) (Table 3). The PO voluntarily chosen and maintained during the first 15 s of exercise was similar on all trials. With reductions in S_P_O_2_, PO progressively decreased across conditions at significantly different rates (P < 0.001). The rate at which PO decreased in FAST and MED was similar, and both had a greater rate of decrease when compared to SLOW (Table 2). During CON, there was no significant change in PO during the trial (INITIAL = 146 ± 66 W; MIDDLE = 149 ± 64 W; FINAL = 146 ± 58 W). Representative data from one subject, illustrating the PO vs. time response, are shown in Fig 1 (bottom).

**Table 3:** Power output (W) at different absolute S_P_O_2_ during constant RPE exercise trials for FAST, MED, and SLOW. The initial power output chosen by participants during CON was 146 ± 66 and remained at this level through out the trial.

	~100%	90%	80%
	S_P_O_2_	S_P_O_2_	S_P_O_2_
FAST	149 ± 68	125 ± 54	94 ± 40^2^
MED	149 ± 67	128 ± 62	99 ± 46^2^
SLOW	146 ± 66	140 ± 66	115 ± 55

Values are means ± SD; n = 12

^2^ Significantly different from SLOW (P < 0.05)

**Fig 2:**
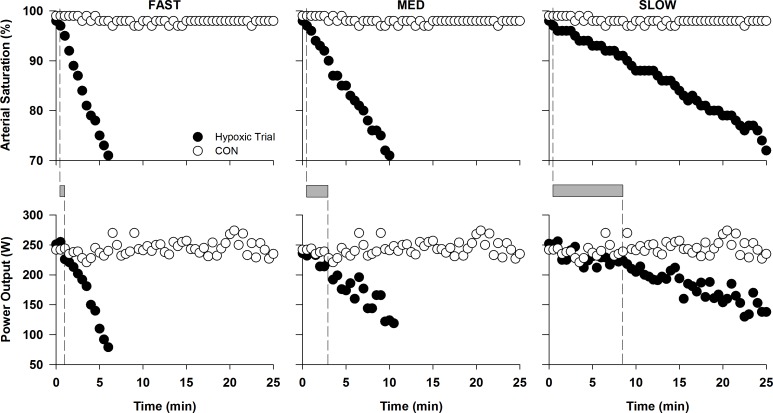
Representative data from one subject illustrating the time delay between the start of arterial desaturation and the subsequent decrease in PO. Dashed lines indicate when FAST, MED, and SLOW began to deviate from CON. The shaded area represents the lag time between the start of arterial desaturation and the ensuing decrease in PO.

Upon the onset of a decrease in S_P_O_2_, the approximate time delay for beginning the decrease in PO was significantly different between each condition (FAST = 0.1 ± 0.2 min; MED = 1.8 ± 1.0 min; SLOW = 5.6 ± 2.3 min; P < 0.05). Representative data from one subject, illustrating the time delay in decreasing PO, are shown in Fig 2.

There was a significant interaction between condition and S_P_O_2_ on PPO (P < 0.001). Post hoc testing revealed that PRE PPO was similar between all of the conditions. During CON, there was a significant decrease in PPO from PRE to POST, however PPO remained at a similar level between POST and REC. When S_P_O_2_ was reduced to 70%, FAST, MED, and SLOW POST PPO significantly decreased to similar levels and all were less than CON. When S_P_O_2_ recovered back to ~ 100% after the constant RPE trials, FAST, MED, and SLOW REC PPO significantly increased from POST and were all similar to CON, but did not recover back to PRE values (Table 4).

**Table 4:** Power output and sEMG_RMS_ responses during the 5 s sprints.

	Stage	FAST	MED	SLOW	CON
PPO (W)	PRE	737 ± 265	733 ± 263	742 ± 268	744 ± 271
	POST	551 ± 204^1^^,^ ^a^	532 ± 188^1^^,^ ^a^	570 ± 214^1^^,^ ^a^	662 ± 248^a^
	REC	685 ± 244^a^^,^ ^b^	661 ± 250^a^^,^ ^b^	669 ± 253^a^^,^ ^b^	668 ± 235^a^
VM sEMG_RMS_ (Δ PRE)	POST	-28 ± 15	-32 ± 11	-23 ± 15	-18 ± 13
	REC	-8 ± 17^b^	- 16 ± 11^b^	-14 ± 13^b^	-15 ± 17
VL sEMG_RMS_ (Δ PRE)	POST	-22 ± 13	-22 ± 11	-19 ± 11	-16 ± 9
	REC	-10 ± 7^b^	-12 ± 11^b^	-13 ± 12^b^	-18 ± 7

Values are means ± SD; PPO n = 12; sEMG_RMS_ n = 10

^1^ Significantly different from CON (P < 0.05)

^a^ Significantly different from PRE (P < 0.05)

^b^ Significantly different from POST (P < 0.05)

### Surface electromyography

With decreases in S_P_O_2_, sEMG_RMS_ decreased at significantly different rates from the beginning of each constant RPE trial in VM (P < 0.001). The FAST and MED were similar, but each had a greater rate of decrease when compared to SLOW. sEMG_RMS_ also decreased at significantly different rates in the VL (P < 0.001). Post hoc testing revealed that the rate of decrease was greater in FAST when compared to SLOW (Table 2). During CON, there was no significant change in sEMG_RMS_ from initial values for either VM (INITIAL = 100 ± 0%; MIDDLE = 103 ± 10%; FINAL = 100 ± 16%) or VL (INITIAL = 100 ± 0%; MIDDLE = 103 ± 11%; FINAL = 99 ± 11%) during the trial.

There was a significant interaction between condition and S_P_O_2_ on sEMG_RMS_ for the VM (P = 0.017) and VL (P = 0.002) during the 5 s sprint. Follow up tests revealed that sEMG_RMS_ values increased from POST to REC during FAST, MED, and SLOW. However, sEMG_RMS_ during CON remained at similar values between POST and REC. Both the VM and VL exhibited these trends (Table 4).

### Tissue oxygenation index

There was a significant interaction between condition and stage on TOI of the VL (P < 0.001) and PFC (P < 0.001). Follow up tests revealed that INITIAL TOI values in the VL were similar across all conditions. With reductions in S_P_O_2_, the decrease in TOI of the VL during FAST, MED, and SLOW was similar and all were less than CON by the end of the trial. Follow up tests also revealed that starting TOI values in the PFC were similar across all conditions. With reductions in S_P_O_2_, the decrease in TOI of the PFC during FAST, MED, and SLOW was similar and all were less than CON by the end of the trial (Table 5).

**Table 5:** NIRS responses at different trial stages during constant RPE exercise.

	Stage	FAST	MED	SLOW	CON
Vastus Lateralis					
TOI (%)	Initial	56 ± 8	58 ± 6	56 ± 9	56 ± 6
	Middle	52 ± 8	53 ± 7	51 ± 9	55 ± 6
	Final	49 ± 8^1^	50 ± 7^1^	48 ± 8^1^	54 ± 7
O_2_Hb (% change from initial)	Initial	-0.2 ± 0.8	0.1 ± 0.9	0.0 ± 0.8	-0.1 ± 0.6
	Middle	-2.7 ± 1.6^1^	-3.2 ± 1.4^1^	-2.9 ± 1.9^1^	0.0 ± 2.2
	Final	-4.7 ± 2.1^1^	-5.1 ± 2.6^1^	-5.4 ±3.2^1^	0.3 ± 2.8
Pre-Frontal Cortex					
TOI (%)	Initial	66 ± 5	66 ± 4	64 ± 6	64 ± 6
	Middle	60 ± 5	61 ± 5	58 ± 7^1^	64 ± 6
	Final	57 ± 6^1^	56 ± 7^1^	53 ± 6^1^	63 ± 7
O_2_Hb (% change from initial)	Initial	-0.1 ± 1.1	0.4 ± 1.4	-0.4 ± 1.2	0.4 ± 0.8
	Middle	-3.8 ± 2.4^1^	-4.2 ± 2.4^1^	-4.2 ± 2.4^1^	2.6 ± 1.9
	Final	-7.5 ± 3.0^1^	-8.7 ± 4.3^1^	-8.1 ± 3.6^1^	3.1 ± 2.9

Values are means ± SD; n = 12

^1^ Significantly different from CON (P < 0.05)

### Oxyhemoglobin

There was a significant interaction between condition and stage on O_2_Hb of the VL (P < 0.001) and PFC (P < 0.001). Follow up tests revealed that starting O_2_Hb values in the VL were similar across all conditions. With reductions in S_P_O_2_, the decrease in O_2_Hb of the VL during FAST, MED, and SLOW was similar and all were less than CON. Follow up tests also revealed that starting O_2_Hb values in the PFC were similar across all conditions. With reductions in S_P_O_2_, the decrease in O_2_Hb of the PFC during FAST, MED, and SLOW was similar. However O_2_Hb during CON increased throughout the trial (Table 5).

### Heart rate

There was a significant interaction between condition and stage on HR (P = 0.001). HR increased with reductions in S_P_O_2_ from 100 to 85% and then remained at a similar value as S_P_O_2_ continued to decrease to 70% for FAST, MED, and SLOW. During CON, HR continuously increased throughout the trial. However, further analysis revealed that starting HR values and the HR response throughout the trials were similar across the four experimental conditions (Table 6).

**Table 6:** Cardiorespiratory responses at different trial stages during constant RPE exercise.

	Stage	FAST	MED	SLOW	CON
HR (BPM)	Initial	141 ± 18	144 ± 19	137 ± 21	143 ± 17
	Middle	146 ± 18	151 ± 17	150 ± 19	147 ± 19
	Final	146 ± 16	153 ± 18	152 ± 20	153 ± 18
${\dot{V}}_{E}$ (L•min^-1^)	Initial	64 ± 21	63 ± 18	60 ± 20	60 ± 17
	Middle	75 ± 26^1^	77 ± 25^1^	75 ± 25^1^	63 ± 20
	Final	78 ± 29^1^	84 ± 24^1^	84 ± 28^1^	65 ± 21
PET_CO2_ (mmHg)	Initial	42 ± 4	42 ± 3	41 ± 3	40 ± 4
	Middle	40 ± 4	42 ± 3	41 ± 3	38 ± 4
	Final	41 ± 4	42 ± 3	41 ± 3	37 ± 4

Values are means ± SD; n = 12

^1^ Significantly different from CON (P < 0.05)

### Minute ventilation

There was a significant interaction between condition and stage on ${\dot{V}}_{E}$ (P < 0.001). Follow up tests revealed that starting ${\dot{V}}_{E}$ values were similar across all conditions. Although ${\dot{V}}_{E}$ increased throughout the trial for each condition, the change in ${\dot{V}}_{E}$ was similar for FAST, MED, and SLOW with decreasing S_P_O_2_ and all were greater than CON (Table 6).

### Partial pressure of end-tidal CO_2_

There was a significant effect of S_P_O_2_ on fresh gas delivery (P < 0.001). Follow up tests revealed that the initial rates of fresh gas delivery at the commencement of the trial and the decrease over the course of the exercise bout were similar for FAST, MED, and SLOW. With decreases in fresh gas delivery, PET_CO2_ values remained within 2 mmHg throughout each trial and in between FAST, MED, and SLOW (Table 6).

## Discussion

This study is the first report of the influence of different rates of change in S_P_O_2_ on self-selected exercise intensity during constant RPE exercise. Despite exercising for less time, reaching a similar absolute change in S_P_O_2_ and achieving a comparable estimated oxygenation status in cerebral and muscle tissues, the relationship between self-selected exercise intensity and muscle activation with S_P_O_2_ was altered, such that a faster arterial deoxygenation rate was associated with a greater decline in submaximal self-selected work rate. These results suggest that the rate of arterial deoxygenation has a central depressant effect on submaximal self-selected exercise intensity, which is independent from the absolute level of S_P_O_2_. In contrast, the decline in PPO, and the associated sEMG, during the 5 s sprint was not affected by the rate of change of S_P_O_2_, suggesting that the magnitude of impairment to the overall capacity of the neuromuscular system was similar.

This investigation corroborates previous research illustrating that hypoxia impairs continuous submaximal exercise performance. Using various exercise modalities, previous research has illustrated that hypoxia increases the time to traverse a given distance [58, 59], and decreases the tolerance time at a given absolute work rate [60, 61]. Several theories of hypoxia-induced fatigue have been proposed that could explain some of the fatigue observed between CON and the experimental conditions. First decreasing brain oxygenation [40] has been reported to affect the ability to sustain a given exercise intensity when S_P_O_2_ is below a critical level of approximately 70% [62]. However, as four of our participants chose to terminate the trial prior to achieving a S_P_O_2_ of 70%, differences in exercise performance across conditions were analyzed when S_P_O_2_ was as high as 80% (Table 3), well above the level that cerebral deoxygenation has been suggested to influence exercise. Second, respiratory muscle demand during continuous high intensity exercise exacerbates the increase in sympathetic vasoconstrictor tone in the exercising limb and contributes to locomotor muscle fatigue by decreasing limb vascular conductance and blood flow [32, 34]. However, ${\dot{V}}_{E}$, and presumably the work of breathing, were similar across the experimental conditions (Table 5) suggesting that their influence on sympathetic vasoconstriction was similar. Furthermore, the work of breathing associated with submaximal exercise intensities similar to this study, did not contribute to the vasoconstrictor activity present in working locomotor muscles [63]. Third, direct recordings from group III and IV afferents from the triceps surae muscle of artificially ventilated, barbiturate-anesthetized cats found that hypoxia (P_a_O_2_ = 23 mmHg) increased the baseline discharge frequency of these afferents at rest as well as potentiated the activity within these afferents during electrically-induced static muscle contraction when compared to normoxia (P_a_O_2_ = 108) [64]. The increase in baseline firing frequency has been suggested to be independent of metabolic changes within the muscle, as the metabolites that stimulate these afferents, such as lactic acid and H^+^, were unaffected when acute hypoxic stimuli were given at rest [64, 65]. Given their prolific innervation of muscle tissue, small changes in their individual discharge rates, would substantially influence their collective input into the CNS [66]. Fourth, a decrease in O_2_ availability decreases $\dot{V}O_{2\max}$ in a dose dependent manner [59, 67], while RPE is reflective of relative physical strain [15]. It stands to reason that with a decrease in S_p_O_2_, $\dot{V}O_{2\max}$ would decrease, thus increasing relative exercise intensity for a given absolute workload. To maintain the same RPE during progressive hypoxia, participants would have to continuously decrease PO to keep their perceived exertion on target.

Although this investigation confirms that a decrease in the absolute magnitude of O_2_ availability impairs submaximal exercise performance, the novel finding of this study is that the impairment to self-selected exercise intensity was magnified when arterial desaturation occurred at a faster rate, at a time when O_2_ availability was similar across conditions (i.e. S_P_O_2_ = 80%). As this study was the first to employ an experimental design to investigate the independent effect of different arterial desaturation rates on self-selected exercise intensity, it is difficult to compare our results with those of previous reports. However, it is unlikely that the alterations to $\dot{V}O_{2\max}$, as well as to the bioenergetic processes within the peripheral tissues, resulting from our hypoxic manipulations had a major differential influence on self-selected exercise intensity and sEMG between FAST, MED, and SLOW when S_P_O_2_ was at 80%. While O_2_ delivery was compromised, O_2_ availability was similar, which should lead to a comparable influence on $\dot{V}O_{2\max}$, tissue aerobic metabolism and subsequently self-selected exercise intensity. As NIRS has been used to directly compare the metabolic state of tissues in various environmental conditions [40], our data illustrate comparable metabolic environments within the respective tissues (Table 4). With respect to the brain, a similar oxygenation status should have similar effects on cerebral metabolism and subsequently CMD generation. With regard to muscle, a comparable metabolic milieu should lead to comparable levels of inhibitory afferent feedback as well as similar effects on muscle metabolism. As the magnitude of tissue deoxygenation was similar between conditions, its direct impact on aerobic metabolism was also likely similar between conditions: i.e. it likely did not have a differential influence on self-selected exercise intensity.

Our data suggest that the generation of self-selected exercise intensity at a constant RPE is sensitive to both the rate of change and absolute magnitude of arterial deoxygenation. The mechanism(s) by which the rate of arterial desaturation modulates the integration of various factors that influence RPE and subsequently the relationship between RPE, self-selected exercise intensity and sEMG remains unknown. However, given the striking similarities in the neuroanatomy involved, the receptors that initiate signals regulating cardiopulmonary function during exercise in hypoxia, may also regulate CMD and muscle activation. Central to this paradigm is the model proposed by Mateika and Duffin [68] highlighting the role of central chemoreceptors, peripheral chemoreceptors, and group III & IV afferents in maintaining a respiratory steady state during exercise. Central chemoreceptors respond to increases in interstitial or CSF H^+^ concentration [69] arising from increasing P_a_CO_2_ levels. As PET_CO2_, and presumably P_a_CO_2_, was maintained within 2 mmHg within each trial and across conditions, central chemoreceptor output should have been controlled, likely not contributing to the modulation of exercise intensity. Based on the available evidence, we can speculate that the metabolic perturbations associated with exercise, combined with the additional stresses of hypoxia, stimulate feedback from either peripheral chemoreceptors and/or group III & IV afferents that project onto and alter the output of the solitary tract nucleus (NTS) [68]. The output from the NTS has several targets including the insular cortex [70]. Williamson *et al*. [71] describe the insular cortex as a likely area involved in the generation of perceived exertion, while Tanaka and Watanabe [72] describe the insular cortex as an area that projects inhibitory signals onto the motor cortex. The hypoxia-induced decrements in self-selected exercise intensity and muscle activation were amplified during constant RPE exercise but not during the 5 s sprint, suggesting that the rate of arterial deoxygenation activates a central adjustment modifying the relationship between RPE and PO rather than alterations in neuromuscular capacity. Although, the proposition that RPE acts as a feed-forward controller has some experimental support [73], its role in feed-forward control remains unclear. However, based on the findings of this investigation, several processes may be postulated that could modulate the physiological and psychological processes involved in the generation of self-selected exercise intensity, which would require further research to confirm.

### Hypothesized mechanisms of homeostatic control

#### Central information processing

As indices of gross respiratory strain, S_P_O_2_, HR, ${\dot{V}}_{E}$, and NIRS parameters were similar between FAST, MED, and SLOW at the end of the trial, the magnitude of the associated afferent signaling was presumably also similar. If confirmed, it would suggest that the CNS independently utilizes the rate of change and absolute magnitude of physiological strain development as concomitant modulators of RPE, CMD, and self-selected exercise intensity. Using computational modeling, Puccini *et al* [74] demonstrated that cortical networks effectively anticipate incoming synaptic inputs by analyzing the magnitude of the stimulus, in combination with its rate of change. Such anticipation supports a proposition that the feed-forward homeostatic control of the neuromuscular system is a function of supraspinal processes.

#### Sensory adaptation

The sensitivity of sensory receptors change in response to the stimulus over time [75]. After reaching a maximum discharge rate, some reports have demonstrated a progressive and significant decline in peripheral chemoreceptor firing activity over time [76] while others have not [77], despite a constant level of hypoxia. Further work is required to determine if sensory adaptation decreases the sensitivity of human peripheral receptors with progressive changes in stimuli over time. Should this theory be confirmed then the attenuated fatigue response during SLOW when compared to FAST and MED could be explained by a decreased level of feedback projecting onto supraspinal centres that alter the generation of RPE and CMD as well as possibly a reduced level of inhibitory feedback projecting onto the MN at the spinal level. In other words, the longer the time required to reach an absolute S_P_O_2_, less feedback would arise to modulate self-selected exercise intensity.

#### Rate sensitive output from peripheral receptors

There are several reviews articles summarizing the stimuli that stimulate central and peripheral chemoreceptors as well as group III & IV afferents [68, 78]. The studies cited in those reviews examined the impact of absolute changes in metabolite concentration on the output of receptors. We are not aware of any investigations that have determined if peripheral receptors are also sensitive to the rate of change in their stimuli. Should this theory be confirmed then the magnified fatigue response during FAST and MED when compared to SLOW could be explained by a greater level of feedback projecting onto supraspinal centres that alter the generation of RPE and CMD as well as possibly a greater level of inhibitory feedback projecting onto the MN at the spinal level. In other words, the faster the rate of physiological strain development, the greater the output from the sensor for a given S_P_O_2_.

#### Spinal modulation

According to Marcora [19], RPE is centrally generated by corollary discharge from motor to sensory areas of the cerebral cortex and not related to afferent feedback from the periphery. This theory has received experimental support in investigations where curare was used to partially block NMJ transmission. With the curare block, the increase in CMD required to exercise at the same workload elicited a greater perceived effort [18]. Accordingly, the decreases in muscle activation during constant RPE exercise could be the result of inhibitory spinal modulation, as CMD would have remained at a similar level. Should this be theory be confirmed then the magnified fatigue response during FAST and MED when compared to SLOW could be explained by either sensory adaptation or a rate sensitive output from the peripheral receptors.

### Limitations

The normal function of the human body during exercise is reliant on the effective integration of a multitude of physiological systems, which we have yet to fully comprehend and appreciate. Although our measures indicated similar amounts of gross respiratory strain across conditions, we did not measure the regional biochemical alterations within the active tissues. For example, the synthesis and release of excitatory neurotransmitters such as acetylcholine [79, 80], aromatic monoamines [79, 81], as well as glutamate and aspartate [82, 83] are reduced during acute hypoxia, while the synthesis and release of the inhibitory neurotransmitters and neuromodulators such as γ-aminobutyric acid (GABA) [83–85], adenosine [86], beta-alanine [82, 83], taurine [87], endogenous opioids [88, 89], and lactate [82, 90] are increased. The shift in the balance between excitatory and inhibitory neuro-effectors would hyperpolarize neuronal membranes, possibly impairing motor function by limiting the generation of CMD. However, given the time required for the body to achieve a cellular steady state during acute hypoxia, the likelihood of neuronal hyperpolarization increases with longer hypoxic exposure, minimizing its influence on the shorter trials. Therefore, if there were differences in the biochemical environment between conditions, the difference in the performance decrement would have likely been underestimated in this study, strengthening the conclusions regarding the independent depressant influence of a faster rate of arterial deoxygenation.

Furthermore, differences in sEMG during constant RPE trials between conditions suggest that central factors contributed to the rate dependent relationship between S_P_O_2_ and self-selected exercise intensity. The underlying assumption is that for a given S_P_O_2_, all else in the O_2_ transport pathway remains similar between conditions. Although the NIRS parameters used in this study suggest that a similar level of cerebral and muscle oxygenation was achieved across conditions, further work is required to confirm this assumption. For example, future work could explore the influence of different deoxygenation rates on the regulation of peripheral factors such as muscle blood flow, blood pressure, and cardiac output.

### Conclusions

When controlling for absolute decreases in S_P_O_2_, a faster rate of arterial deoxygenation was associated with a greater decrement in self-selected exercise intensity than a slower rate when exercising at a RPE of 5 on Borg’s 10-point scale but not on PPO during a 5 s maximal effort sprint. This decrement in performance was accompanied by a great reduction in muscle activation, suggesting that central processes were involved. Whether the rate sensitive component within the proposed framework is a function of central information processing leading to changes in CMD or the modulation of CMD at the spinal level, does not conceptually change the feed-forward model as each possibility allow the organism to differentiate between the instantaneous state and the instantaneous rate of change [13]. Further research should be undertaken to clarify if peripheral factors contributed to these observations, if the regulation of exercise intensity is similarly sensitive to the rate of change of other physiological stimuli, and to elucidate the mechanisms responsible for mediating this effect.
